# Phenyl­hydrazinium (6-carb­oxy­pyridine-2-carboxyl­ato)(pyridine-2,6-dicarboxyl­ato)cobaltate(II)–pyridine-2,6-dicarb­oxy­lic acid–water (1/1/3)

**DOI:** 10.1107/S1600536810048191

**Published:** 2010-11-24

**Authors:** Consuelo Yuste, Manuela Ramos Silva, Mohammad Ghadermazi, Fariba Feizi, Elham Motieiyan

**Affiliations:** a CEMDRX, Physics Department, University of Coimbra, P-3004-516 Coimbra, Portugal; b Department of Chemistry, Faculty of Science, University of Kurdistan, Sanandaj, Iran; c Department of Chemistry, Faculty of Science, Payame Noor University, Qom, Iran

## Abstract

The asymmetric unit of the title compound, (C_6_H_9_N_2_)[Co(C_7_H_3_NO_4_)(C_7_H_4_NO_4_)]·C_7_H_5_NO_4_·3H_2_O, contains one (6-carb­oxy­pyridine-2-carboxyl­ato)(pyridine-2,6-dicarboxyl­ato)cobaltate(II) anion, one phenyl­hydrazinium cation, one pyridine-2,6-dicarb­oxy­lic acid mol­ecule and three uncoordin­ated water mol­ecules, part of which are disordered. The Co^II^ ion is coordinated by a pyridine-2,6-dicarboxyl­ate ion and a 6-carb­oxy­pyridine-2-carboxyl­ate ligand almost perpendicular to each other [the angle between the least-squares planes is 87.38 (4)°] and is surrounded by two O atoms and two N atoms in the equatorial plane and two O atoms in axial positions, resulting in a distorted octa­hedral coordination geometry. There is an extensive three-dimensional network of O—H⋯O and N—H⋯O hydrogen bonds, which link the components.

## Related literature

For related cobalt, copper and cadmium complexes containing 2,6-dicarboxyl­ato ligands, see: Aghabozorg *et al.* (2008 ▶); Aghabozorg *et al.* (2009 ▶); Moghimi *et al.* (2002 ▶). For an isotypic series of five related *M*(II) complexes, see: MacDonald *et al.* (2004 ▶). For the supra­molecular chemistry and crystal structures of five bis­(imidazolium 2,6-pyridine­dicarboxyl­ate)*M*(II) complexes, see: MacDonald *et al.* (2000 ▶).
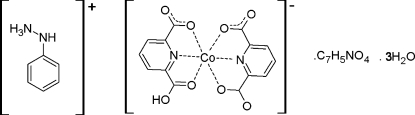


## Experimental

### 

#### Crystal data

(C_6_H_9_N_2_)[Co(C_7_H_3_NO_4_)(C_7_H_4_NO_4_)]·C_7_H_5_NO_4_·3H_2_O
*M* *_r_* = 720.47
Triclinic, 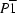

*a* = 8.8019 (4) Å
*b* = 12.2378 (5) Å
*c* = 14.6559 (7) Å
α = 101.080 (2)°
β = 91.351 (3)°
γ = 98.749 (3)°
*V* = 1528.95 (12) Å^3^
*Z* = 2
Mo *K*α radiation
μ = 0.64 mm^−1^
*T* = 293 K
0.25 × 0.12 × 0.12 mm

#### Data collection

Bruker APEXII CCD area-detector diffractometer
Absorption correction: multi-scan (*SADABS*; Sheldrick, 2000 ▶) *T*_min_ = 0.825, *T*_max_ = 0.999
27004 measured reflections
5526 independent reflections
4198 reflections with *I* > 2σ(*I*)
*R*_int_ = 0.038

#### Refinement

*R*[*F* ^2^ > 2σ(*F* ^2^)] = 0.043
*wR*(*F* ^2^) = 0.167
*S* = 1.13
5526 reflections
462 parameters
6 restraints
H atoms treated by a mixture of independent and constrained refinement
Δρ_max_ = 0.71 e Å^−3^
Δρ_min_ = −0.78 e Å^−3^

### 

Data collection: *APEX2* (Bruker, 2003 ▶); cell refinement: *SAINT* (Bruker, 2003 ▶); data reduction: *SAINT*; program(s) used to solve structure: *SHELXS97* (Sheldrick, 2008 ▶); program(s) used to refine structure: *SHELXL97* (Sheldrick, 2008 ▶); molecular graphics: *ORTEPII* (Johnson, 1976 ▶); software used to prepare material for publication: *SHELXL97*.

## Supplementary Material

Crystal structure: contains datablocks global, I. DOI: 10.1107/S1600536810048191/si2309sup1.cif

Structure factors: contains datablocks I. DOI: 10.1107/S1600536810048191/si2309Isup2.hkl

Additional supplementary materials:  crystallographic information; 3D view; checkCIF report

## Figures and Tables

**Table 1:** Selected bond lengths (Å)

Co1—N1	2.017 (2)
Co1—N2	2.033 (2)
Co1—O5	2.090 (2)
Co1—O1	2.148 (2)
Co1—O3	2.175 (2)
Co1—O7	2.281 (2)

**Table 2:** Hydrogen-bond geometry (Å, °)

*D*—H⋯*A*	*D*—H	H⋯*A*	*D*⋯*A*	*D*—H⋯*A*
O8—H8⋯O14	0.82	1.67	2.491 (4)	174
O13—H13*A*⋯O6^i^	0.85 (2)	2.29 (2)	2.917 (4)	131 (3)
O13—H13*B*⋯O4^ii^	0.85 (3)	2.09 (2)	2.926 (4)	168 (3)
O14—H14*A*⋯O4^ii^	0.85 (2)	1.80 (2)	2.645 (4)	174 (3)
O14—H14*B*⋯O15	0.85 (3)	1.98 (4)	2.658 (6)	136 (4)
O9—H9⋯O2^iii^	0.82	1.70	2.520 (3)	173
O11—H11*A*⋯O13^iv^	0.82	1.84	2.634 (4)	163
N4—H4*A*⋯O6^i^	0.89	2.06	2.935 (3)	167
N4—H4*A*⋯O5^i^	0.89	2.53	2.993 (3)	113
N4—H4*A*⋯O11^v^	0.89	2.58	3.011 (3)	110
N4—H4*B*⋯O10^vi^	0.89	2.05	2.834 (3)	146
N4—H4*C*⋯O9^v^	0.89	2.41	2.964 (3)	121
N5—H5*A*⋯O1^vii^	0.99 (3)	2.12 (3)	3.060 (3)	158 (3)

Symmetry codes: (i) 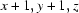; (ii) 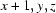; (iii) 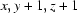; (iv) 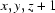; (v) 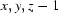; (vi) 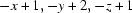; (vii) 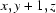.

**Fig. 1.**
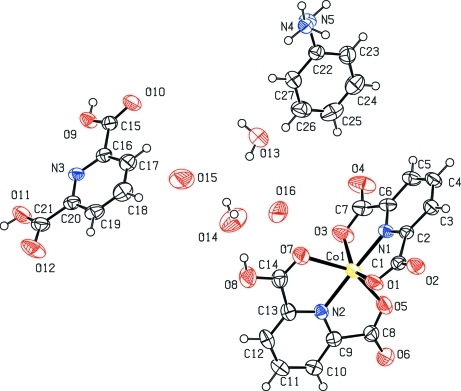
ORTEPII (Johnson, 1976) plot of the title compound. Displacement ellipsoids are drawn at the 50% level.

**Fig. 2.**
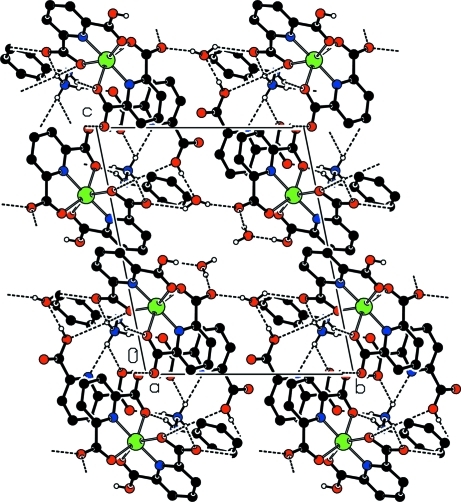
Packing of the molecules in the unit cell showing the H-bonds as dashed lines. H atoms not involved in H-bonding were omitted for clarity. Also omitted were the disordered water O atoms.

## supplementary crystallographic information

### Comment

Many complexes containing pyridine-2,6-dicarboxylate, Co^II^ ions and various bases have been reported (Aghabozorg *et al.* 2008, 2009; Moghimi *et al.*, 2002; MacDonald *et al.*, 2004, 2000).

In the title compound (I), Fig. 1, the metal ion Co^II^ is six-coordinated by two pyridine-2,6-dicarboxylate ligands, with -2 and -1 negative charges (Table 1). Both ligands are tridentate and the coordination sphere around the cobalt is a distorted octahedral with the N—Co—N angle equal to 177.68 (9)°. The angle between the least-squares plane of the non-H atoms of the two ligands is 87.38 (4)°. Each complex of total charge -1 is accompanied in the asymmetric unit cell by one phenylhydrazinium ion as a counter ion, a neutral pyridine-2,6-dicarboxylic acid molecule and three water molecules. In the cation the terminal N4 is deviated by 1.188 (3)Å from the C22—C27 plane. The bond angle sum around N5 is 327°, indicative of a *sp*^3^ hybridization for this atom. There is an extensive three-dimensional network of H-bonds linking the molecules and ions together. Water O13 links the cobalt complexes along the *b* axis and water O14 links the cobalt complexes along the *a* axis. The neutral acidic molecule is H-bonded to the complex and the cation shares the NH and NH_3_ hydrogen atoms with two symmetry related complexes and the neutral acid molecule (Table 2 and Fig. 2).

### Experimental

From a solution of phenylhydrazine (0.4 mmol) and of pyridine-2,6-dicarboxylic acid (0.4 mmol) in THF (30 ml), a white precipitate was obtained. By mixing the precipitate with cobalt (II) nitrate (0.2 mmol) in water (25 ml), brown crystals of the title compound were obtained after allowing the mixture to stand for 2 weeks at room temperature.

### Refinement

The occupancy of water O atoms, O15 and O16, refined to near 50%, so that at the final stages of refinement the sum of their occupancies was set to one. H atoms bound to C atoms were placed at calculated positions and were treated as riding on the parent atoms with C—H = 0.93 Å (aromatic) and 0.98 Å (CH) and with *U*_iso_(H) = 1.2 *U*_eq_(C). H atoms of water molecules O13 an O14 were located in a difference Fourier map and refined as riding with O—H = 0.85 (1) Å, H—H = 1.34 (1) Å and *U*_iso_(H) = 1.5Ueq(O). The H atoms of the remaining water molecule disordered over two sites could not be located. H atoms of hydroxyl and hydrazinium groups were located in a difference electron density map but O–H were refined using AFIX 147 and NH_3_ H atoms using AFIX 137 in *SHELXL97*. The coordinates of H atom attached to N5 were freely refined, *U*_iso_(H5) = 1.2 *U*_eq_(N5).

### Crystal data

**Table:**

(C_6_H_9_N_2_)[Co(C_7_H_3_NO_4_)(C_7_H_4_NO_4_)]·C_7_H_5_NO_4_·3H_2_O	*Z* = 2
*M**_r_* = 720.47	*F*(000) = 742
Triclinic, *P*1	*D*_x_ = 1.565 Mg m^−^^3^
*a* = 8.8019 (4) Å	Mo *K*α radiation, λ = 0.71073 Å
*b* = 12.2378 (5) Å	Cell parameters from 8005 reflections
*c* = 14.6559 (7) Å	θ = 2.8–25.2°
α = 101.080 (2)°	µ = 0.64 mm^−^^1^
β = 91.351 (3)°	*T* = 293 K
γ = 98.749 (3)°	Block, brown
*V* = 1528.95 (12) Å^3^	0.25 × 0.12 × 0.12 mm

### Data collection

**Table:**

Bruker APEX CCD area-detector diffractometer	5526 independent reflections
Radiation source: fine-focus sealed tube	4198 reflections with *I* > 2σ(*I*)
graphite	*R*_int_ = 0.038
φ and ω scans	θ_max_ = 25.3°, θ_min_ = 1.7°
Absorption correction: multi-scan (*SADABS*; Sheldrick, 2000)	*h* = −10→10
*T*_min_ = 0.825, *T*_max_ = 0.999	*k* = −14→14
27004 measured reflections	*l* = −17→17

### Refinement

**Table:**

Refinement on *F*^2^	Primary atom site location: structure-invariant direct methods
Least-squares matrix: full	Secondary atom site location: difference Fourier map
*R*[*F*^2^ > 2σ(*F*^2^)] = 0.043	Hydrogen site location: inferred from neighbouring sites
*wR*(*F*^2^) = 0.167	H atoms treated by a mixture of independent and constrained refinement
*S* = 1.13	*w* = 1/[σ^2^(*F*_o_^2^) + (0.1037*P*)^2^] where *P* = (*F*_o_^2^ + 2*F*_c_^2^)/3
5526 reflections	(Δ/σ)_max_ = 0.002
462 parameters	Δρ_max_ = 0.71 e Å^−^^3^
6 restraints	Δρ_min_ = −0.78 e Å^−^^3^

### Special details

**Table:**

Geometry. All e.s.d.'s (except the e.s.d. in the dihedral angle between two l.s. planes) are estimated using the full covariance matrix. The cell e.s.d.'s are taken into account individually in the estimation of e.s.d.'s in distances, angles and torsion angles; correlations between e.s.d.'s in cell parameters are only used when they are defined by crystal symmetry. An approximate (isotropic) treatment of cell e.s.d.'s is used for estimating e.s.d.'s involving l.s. planes.
Refinement. Refinement of *F*^2^ against ALL reflections. The weighted *R*-factor *wR* and goodness of fit *S* are based on *F*^2^, conventional *R*-factors *R* are based on *F*, with *F* set to zero for negative *F*^2^. The threshold expression of *F*^2^ > σ(*F*^2^) is used only for calculating *R*-factors(gt) *etc*. and is not relevant to the choice of reflections for refinement. *R*-factors based on *F*^2^ are statistically about twice as large as those based on *F*, and *R*- factors based on ALL data will be even larger.

### Fractional atomic coordinates and isotropic or equivalent isotropic displacement parameters (Å^2^)

**Table:**

	*x*	*y*	*z*	*U*_iso_*/*U*_eq_	Occ. (<1)
Co1	0.08241 (5)	0.11526 (3)	0.27416 (3)	0.03986 (15)	
N1	0.0353 (3)	0.20143 (19)	0.17546 (17)	0.0321 (5)	
N2	0.1315 (3)	0.0232 (2)	0.36950 (16)	0.0333 (6)	
O1	0.1988 (3)	0.04183 (18)	0.15618 (15)	0.0439 (5)	
O2	0.2202 (3)	0.03896 (18)	0.00472 (16)	0.0467 (6)	
O3	−0.0290 (3)	0.2509 (2)	0.34757 (17)	0.0604 (7)	
O4	−0.1504 (3)	0.3913 (2)	0.32449 (19)	0.0718 (8)	
O5	−0.1078 (3)	−0.0133 (2)	0.25722 (17)	0.0549 (6)	
O6	−0.2095 (3)	−0.1669 (2)	0.30695 (18)	0.0554 (6)	
O7	0.3082 (3)	0.2062 (2)	0.35125 (17)	0.0533 (6)	
O8	0.4774 (3)	0.1909 (2)	0.46295 (19)	0.0645 (7)	
H8	0.5193	0.2522	0.4538	0.097*	
O13	0.7224 (4)	0.5896 (2)	0.2939 (2)	0.0735 (8)	
H13A	0.767 (4)	0.6505 (12)	0.328 (2)	0.110*	
H13B	0.765 (3)	0.5383 (15)	0.310 (3)	0.110*	
O14	0.6225 (4)	0.3733 (3)	0.4384 (2)	0.0911 (10)	
H14A	0.696 (2)	0.384 (3)	0.4033 (16)	0.137*	
H14B	0.606 (4)	0.4388 (13)	0.464 (3)	0.137*	
O15	0.7032 (9)	0.5579 (4)	0.5692 (4)	0.111 (3)	0.600 (6)
O16	0.0711 (12)	0.3917 (6)	0.5180 (5)	0.100 (4)	0.400 (6)
C1	0.1723 (3)	0.0746 (2)	0.0823 (2)	0.0344 (7)	
C2	0.0767 (3)	0.1683 (2)	0.0894 (2)	0.0315 (6)	
C3	0.0373 (4)	0.2194 (3)	0.0181 (2)	0.0388 (7)	
H3	0.0651	0.1947	−0.0424	0.047*	
C4	−0.0448 (4)	0.3087 (3)	0.0388 (2)	0.0430 (8)	
H4	−0.0725	0.3448	−0.0079	0.052*	
C5	−0.0851 (4)	0.3435 (3)	0.1288 (2)	0.0437 (8)	
H5	−0.1393	0.4035	0.1437	0.052*	
C6	−0.0434 (3)	0.2873 (2)	0.1966 (2)	0.0355 (7)	
C7	−0.0777 (4)	0.3123 (3)	0.2975 (2)	0.0472 (8)	
C8	−0.1070 (4)	−0.0855 (3)	0.3071 (2)	0.0413 (8)	
C9	0.0315 (3)	−0.0679 (2)	0.3749 (2)	0.0357 (7)	
C10	0.0559 (4)	−0.1344 (3)	0.4379 (2)	0.0417 (8)	
H10	−0.0138	−0.1988	0.4404	0.050*	
C11	0.1854 (4)	−0.1037 (3)	0.4969 (2)	0.0465 (8)	
H11	0.2037	−0.1474	0.5401	0.056*	
C12	0.2878 (4)	−0.0085 (3)	0.4920 (2)	0.0438 (8)	
H12	0.3755	0.0137	0.5318	0.053*	
C13	0.2571 (3)	0.0526 (3)	0.4267 (2)	0.0388 (7)	
C14	0.3519 (4)	0.1574 (3)	0.4096 (2)	0.0441 (8)	
N3	0.5452 (3)	0.71742 (19)	0.98841 (17)	0.0336 (6)	
O9	0.3803 (3)	0.88505 (18)	1.00552 (14)	0.0423 (5)	
H9	0.3352	0.9391	1.0061	0.063*	
O10	0.3851 (3)	0.88449 (19)	0.85355 (16)	0.0494 (6)	
O11	0.6360 (3)	0.6510 (2)	1.14056 (17)	0.0620 (7)	
H11A	0.6807	0.6353	1.1849	0.093*	
O12	0.7558 (3)	0.5215 (2)	1.0602 (2)	0.0749 (8)	
C16	0.4987 (3)	0.7465 (2)	0.9112 (2)	0.0354 (7)	
C17	0.5231 (4)	0.6919 (3)	0.8224 (2)	0.0458 (8)	
H17	0.4890	0.7164	0.7704	0.055*	
C18	0.5987 (4)	0.6008 (3)	0.8129 (3)	0.0550 (9)	
H18	0.6161	0.5617	0.7541	0.066*	
C19	0.6486 (4)	0.5682 (3)	0.8917 (3)	0.0475 (8)	
H19	0.7006	0.5068	0.8872	0.057*	
C20	0.6203 (3)	0.6280 (2)	0.9773 (2)	0.0378 (7)	
C15	0.4154 (3)	0.8466 (2)	0.9206 (2)	0.0353 (7)	
C21	0.6782 (4)	0.5948 (3)	1.0634 (3)	0.0456 (8)	
N4	0.5745 (3)	0.8905 (2)	0.17482 (17)	0.0379 (6)	
H4A	0.6351	0.8616	0.2101	0.057*	
H4B	0.6256	0.9537	0.1619	0.057*	
H4C	0.5459	0.8410	0.1221	0.057*	
N5	0.4388 (3)	0.9152 (2)	0.22451 (19)	0.0416 (6)	
H5A	0.373 (4)	0.950 (3)	0.186 (2)	0.050*	
C22	0.3461 (4)	0.8152 (2)	0.2400 (2)	0.0367 (7)	
C23	0.1992 (4)	0.7818 (3)	0.1991 (2)	0.0488 (9)	
H23	0.1624	0.8224	0.1582	0.059*	
C24	0.1081 (4)	0.6894 (3)	0.2185 (3)	0.0580 (10)	
H24	0.0091	0.6677	0.1910	0.070*	
C25	0.1611 (5)	0.6277 (3)	0.2787 (3)	0.0599 (11)	
H25	0.0990	0.5644	0.2913	0.072*	
C26	0.3070 (5)	0.6615 (3)	0.3196 (3)	0.0566 (10)	
H26	0.3432	0.6208	0.3606	0.068*	
C27	0.4004 (4)	0.7545 (3)	0.3010 (2)	0.0483 (8)	
H27	0.4991	0.7765	0.3290	0.058*	

### Atomic displacement parameters (Å^2^)

**Table:**

	*U*^11^	*U*^22^	*U*^33^	*U*^12^	*U*^13^	*U*^23^
Co1	0.0464 (3)	0.0381 (2)	0.0397 (3)	0.0132 (2)	0.0000 (2)	0.01435 (19)
N1	0.0308 (12)	0.0296 (12)	0.0389 (14)	0.0099 (10)	0.0025 (11)	0.0103 (10)
N2	0.0325 (13)	0.0362 (13)	0.0320 (13)	0.0088 (11)	0.0009 (11)	0.0062 (10)
O1	0.0504 (13)	0.0434 (12)	0.0459 (13)	0.0248 (10)	0.0005 (10)	0.0152 (10)
O2	0.0571 (14)	0.0433 (12)	0.0446 (13)	0.0251 (11)	0.0051 (11)	0.0068 (10)
O3	0.0909 (19)	0.0574 (15)	0.0440 (14)	0.0353 (14)	0.0181 (13)	0.0174 (12)
O4	0.104 (2)	0.0577 (15)	0.0709 (18)	0.0476 (15)	0.0441 (16)	0.0228 (13)
O5	0.0472 (14)	0.0600 (15)	0.0607 (15)	0.0037 (12)	−0.0168 (12)	0.0258 (13)
O6	0.0474 (14)	0.0505 (14)	0.0652 (16)	−0.0032 (12)	−0.0096 (12)	0.0138 (12)
O7	0.0584 (15)	0.0524 (14)	0.0492 (14)	−0.0025 (12)	−0.0069 (12)	0.0202 (12)
O8	0.0483 (15)	0.0739 (19)	0.0680 (17)	−0.0122 (13)	−0.0157 (13)	0.0254 (15)
O13	0.090 (2)	0.0642 (17)	0.0686 (19)	0.0135 (16)	−0.0180 (16)	0.0220 (15)
O14	0.097 (2)	0.076 (2)	0.088 (2)	−0.0165 (18)	0.0360 (19)	0.0076 (18)
O15	0.196 (7)	0.056 (3)	0.082 (4)	0.050 (4)	−0.017 (4)	−0.006 (3)
O16	0.175 (9)	0.061 (5)	0.050 (5)	−0.001 (5)	−0.032 (5)	−0.002 (4)
C1	0.0299 (15)	0.0291 (15)	0.0440 (18)	0.0072 (12)	−0.0010 (13)	0.0050 (13)
C2	0.0296 (14)	0.0272 (14)	0.0377 (17)	0.0064 (12)	−0.0018 (12)	0.0055 (12)
C3	0.0424 (17)	0.0395 (17)	0.0370 (17)	0.0112 (14)	0.0000 (14)	0.0102 (14)
C4	0.0484 (19)	0.0414 (17)	0.0454 (19)	0.0167 (15)	−0.0020 (15)	0.0170 (15)
C5	0.0425 (18)	0.0367 (16)	0.058 (2)	0.0165 (14)	0.0052 (16)	0.0165 (15)
C6	0.0357 (16)	0.0267 (14)	0.0470 (18)	0.0088 (12)	0.0075 (14)	0.0111 (13)
C7	0.057 (2)	0.0402 (18)	0.050 (2)	0.0163 (16)	0.0156 (17)	0.0155 (15)
C8	0.0385 (17)	0.0402 (18)	0.0445 (19)	0.0089 (15)	−0.0032 (14)	0.0055 (15)
C9	0.0366 (16)	0.0356 (16)	0.0352 (16)	0.0121 (13)	0.0035 (13)	0.0028 (13)
C10	0.0446 (18)	0.0393 (17)	0.0446 (19)	0.0113 (14)	0.0047 (15)	0.0126 (14)
C11	0.0488 (19)	0.053 (2)	0.0465 (19)	0.0175 (16)	0.0058 (16)	0.0241 (16)
C12	0.0386 (17)	0.062 (2)	0.0354 (17)	0.0181 (16)	−0.0019 (14)	0.0139 (15)
C13	0.0322 (16)	0.0497 (18)	0.0360 (17)	0.0120 (14)	0.0028 (13)	0.0081 (14)
C14	0.0376 (18)	0.052 (2)	0.0418 (19)	0.0032 (15)	0.0008 (15)	0.0095 (16)
N3	0.0318 (13)	0.0267 (12)	0.0436 (15)	0.0073 (10)	0.0022 (11)	0.0081 (10)
O9	0.0508 (13)	0.0435 (12)	0.0390 (12)	0.0241 (10)	0.0050 (10)	0.0109 (10)
O10	0.0688 (16)	0.0441 (13)	0.0418 (13)	0.0214 (12)	0.0017 (11)	0.0150 (10)
O11	0.0911 (19)	0.0541 (14)	0.0491 (15)	0.0348 (14)	−0.0068 (14)	0.0137 (12)
O12	0.090 (2)	0.0721 (17)	0.083 (2)	0.0550 (16)	0.0105 (16)	0.0316 (15)
C16	0.0360 (16)	0.0297 (15)	0.0401 (17)	0.0055 (13)	0.0023 (13)	0.0058 (13)
C17	0.053 (2)	0.0431 (18)	0.0412 (19)	0.0128 (16)	0.0045 (16)	0.0041 (15)
C18	0.065 (2)	0.049 (2)	0.049 (2)	0.0212 (18)	0.0111 (18)	−0.0050 (17)
C19	0.0490 (19)	0.0331 (16)	0.060 (2)	0.0140 (15)	0.0082 (17)	0.0027 (15)
C20	0.0343 (16)	0.0282 (15)	0.0509 (19)	0.0073 (13)	0.0026 (14)	0.0063 (13)
C15	0.0331 (16)	0.0316 (15)	0.0415 (18)	0.0046 (13)	0.0010 (13)	0.0085 (13)
C21	0.0457 (19)	0.0328 (16)	0.062 (2)	0.0110 (15)	0.0006 (16)	0.0145 (15)
N4	0.0375 (14)	0.0356 (13)	0.0411 (15)	0.0069 (11)	−0.0027 (11)	0.0089 (11)
N5	0.0447 (15)	0.0362 (14)	0.0466 (16)	0.0124 (12)	0.0043 (13)	0.0095 (12)
C22	0.0430 (17)	0.0337 (16)	0.0337 (16)	0.0103 (14)	0.0060 (14)	0.0032 (13)
C23	0.048 (2)	0.049 (2)	0.048 (2)	0.0101 (17)	−0.0021 (16)	0.0062 (16)
C24	0.048 (2)	0.056 (2)	0.065 (3)	0.0004 (18)	0.0050 (18)	0.0026 (19)
C25	0.072 (3)	0.041 (2)	0.061 (2)	−0.0036 (19)	0.025 (2)	0.0023 (18)
C26	0.081 (3)	0.046 (2)	0.046 (2)	0.014 (2)	0.0116 (19)	0.0153 (16)
C27	0.055 (2)	0.049 (2)	0.0430 (19)	0.0095 (17)	−0.0043 (16)	0.0129 (16)

### Geometric parameters (Å, °)

**Table:**

Co1—N1	2.017 (2)	C12—C13	1.368 (4)
Co1—N2	2.033 (2)	C12—H12	0.9300
Co1—O5	2.090 (2)	C13—C14	1.487 (5)
Co1—O1	2.148 (2)	N3—C16	1.323 (4)
Co1—O3	2.175 (2)	N3—C20	1.348 (3)
Co1—O7	2.281 (2)	O9—C15	1.304 (4)
N1—C2	1.325 (4)	O9—H9	0.8200
N1—C6	1.337 (3)	O10—C15	1.206 (4)
N2—C9	1.327 (4)	O11—C21	1.300 (4)
N2—C13	1.332 (4)	O11—H11A	0.8200
O1—C1	1.254 (4)	O12—C21	1.202 (4)
O2—C1	1.244 (4)	C16—C17	1.382 (4)
O3—C7	1.257 (4)	C16—C15	1.507 (4)
O4—C7	1.247 (4)	C17—C18	1.369 (4)
O5—C8	1.251 (4)	C17—H17	0.9300
O6—C8	1.238 (4)	C18—C19	1.376 (5)
O7—C14	1.219 (4)	C18—H18	0.9300
O8—C14	1.303 (4)	C19—C20	1.375 (4)
O8—H8	0.8200	C19—H19	0.9300
O13—H13A	0.85 (2)	C20—C21	1.498 (4)
O13—H13B	0.85 (3)	N4—N5	1.454 (3)
O14—H14A	0.85 (2)	N4—H4A	0.8900
O14—H14B	0.85 (3)	N4—H4B	0.8900
C1—C2	1.512 (4)	N4—H4C	0.8900
C2—C3	1.380 (4)	N5—C22	1.424 (4)
C3—C4	1.390 (4)	N5—H5A	0.99 (3)
C3—H3	0.9300	C22—C23	1.380 (5)
C4—C5	1.377 (5)	C22—C27	1.386 (4)
C4—H4	0.9300	C23—C24	1.365 (5)
C5—C6	1.385 (4)	C23—H23	0.9300
C5—H5	0.9300	C24—C25	1.382 (5)
C6—C7	1.500 (4)	C24—H24	0.9300
C8—C9	1.517 (4)	C25—C26	1.373 (6)
C9—C10	1.375 (4)	C25—H25	0.9300
C10—C11	1.373 (5)	C26—C27	1.376 (5)
C10—H10	0.9300	C26—H26	0.9300
C11—C12	1.376 (5)	C27—H27	0.9300
C11—H11	0.9300		
			
N1—Co1—N2	177.68 (9)	C13—C12—C11	118.1 (3)
N1—Co1—O5	102.10 (9)	C13—C12—H12	121.0
N2—Co1—O5	77.14 (9)	C11—C12—H12	121.0
N1—Co1—O1	76.28 (8)	N2—C13—C12	122.1 (3)
N2—Co1—O1	101.57 (8)	N2—C13—C14	111.1 (3)
O5—Co1—O1	96.18 (10)	C12—C13—C14	126.8 (3)
N1—Co1—O3	75.72 (9)	O7—C14—O8	125.3 (3)
N2—Co1—O3	106.53 (9)	O7—C14—C13	120.2 (3)
O5—Co1—O3	97.68 (10)	O8—C14—C13	114.5 (3)
O1—Co1—O3	150.78 (9)	C16—N3—C20	116.3 (3)
N1—Co1—O7	107.38 (9)	C15—O9—H9	109.5
N2—Co1—O7	73.46 (9)	C21—O11—H11A	109.5
O5—Co1—O7	150.47 (9)	N3—C16—C17	124.3 (3)
O1—Co1—O7	92.35 (9)	N3—C16—C15	118.0 (3)
O3—Co1—O7	88.12 (10)	C17—C16—C15	117.7 (3)
C2—N1—C6	121.0 (2)	C18—C17—C16	118.3 (3)
C2—N1—Co1	119.22 (18)	C18—C17—H17	120.8
C6—N1—Co1	119.7 (2)	C16—C17—H17	120.8
C9—N2—C13	119.9 (3)	C17—C18—C19	118.9 (3)
C9—N2—Co1	117.7 (2)	C17—C18—H18	120.5
C13—N2—Co1	122.4 (2)	C19—C18—H18	120.5
C1—O1—Co1	115.10 (17)	C20—C19—C18	118.8 (3)
C7—O3—Co1	115.3 (2)	C20—C19—H19	120.6
C8—O5—Co1	116.8 (2)	C18—C19—H19	120.6
C14—O7—Co1	112.8 (2)	N3—C20—C19	123.3 (3)
C14—O8—H8	109.5	N3—C20—C21	117.7 (3)
H13A—O13—H13B	105 (3)	C19—C20—C21	119.0 (3)
H14A—O14—H14B	105 (3)	O10—C15—O9	125.0 (3)
O2—C1—O1	126.1 (3)	O10—C15—C16	120.9 (3)
O2—C1—C2	117.5 (3)	O9—C15—C16	114.1 (3)
O1—C1—C2	116.4 (3)	O12—C21—O11	123.5 (3)
N1—C2—C3	121.4 (3)	O12—C21—C20	122.3 (3)
N1—C2—C1	112.3 (2)	O11—C21—C20	114.2 (3)
C3—C2—C1	126.4 (3)	N5—N4—H4A	109.5
C2—C3—C4	118.3 (3)	N5—N4—H4B	109.5
C2—C3—H3	120.9	H4A—N4—H4B	109.5
C4—C3—H3	120.9	N5—N4—H4C	109.5
C5—C4—C3	119.8 (3)	H4A—N4—H4C	109.5
C5—C4—H4	120.1	H4B—N4—H4C	109.5
C3—C4—H4	120.1	C22—N5—N4	111.7 (2)
C4—C5—C6	118.7 (3)	C22—N5—H5A	106 (2)
C4—C5—H5	120.7	N4—N5—H5A	109.6 (19)
C6—C5—H5	120.7	C23—C22—C27	119.7 (3)
N1—C6—C5	120.8 (3)	C23—C22—N5	120.5 (3)
N1—C6—C7	112.8 (2)	C27—C22—N5	119.6 (3)
C5—C6—C7	126.4 (3)	C24—C23—C22	120.1 (3)
O4—C7—O3	125.9 (3)	C24—C23—H23	120.0
O4—C7—C6	117.9 (3)	C22—C23—H23	120.0
O3—C7—C6	116.2 (3)	C23—C24—C25	120.8 (4)
O6—C8—O5	125.9 (3)	C23—C24—H24	119.6
O6—C8—C9	118.3 (3)	C25—C24—H24	119.6
O5—C8—C9	115.8 (3)	C26—C25—C24	118.9 (4)
N2—C9—C10	121.3 (3)	C26—C25—H25	120.5
N2—C9—C8	112.5 (3)	C24—C25—H25	120.5
C10—C9—C8	126.2 (3)	C25—C26—C27	121.1 (4)
C11—C10—C9	118.7 (3)	C25—C26—H26	119.4
C11—C10—H10	120.7	C27—C26—H26	119.4
C9—C10—H10	120.7	C26—C27—C22	119.3 (3)
C10—C11—C12	120.0 (3)	C26—C27—H27	120.3
C10—C11—H11	120.0	C22—C27—H27	120.3
C12—C11—H11	120.0		
			
N2—Co1—N1—C2	−15 (2)	Co1—O3—C7—O4	176.8 (3)
O5—Co1—N1—C2	−86.1 (2)	Co1—O3—C7—C6	−3.6 (4)
O1—Co1—N1—C2	7.4 (2)	N1—C6—C7—O4	179.8 (3)
O3—Co1—N1—C2	178.9 (2)	C5—C6—C7—O4	−0.3 (5)
O7—Co1—N1—C2	95.6 (2)	N1—C6—C7—O3	0.1 (4)
N2—Co1—N1—C6	161 (2)	C5—C6—C7—O3	−180.0 (3)
O5—Co1—N1—C6	90.7 (2)	Co1—O5—C8—O6	179.7 (3)
O1—Co1—N1—C6	−175.8 (2)	Co1—O5—C8—C9	1.3 (4)
O3—Co1—N1—C6	−4.3 (2)	C13—N2—C9—C10	−1.0 (4)
O7—Co1—N1—C6	−87.7 (2)	Co1—N2—C9—C10	−179.7 (2)
N1—Co1—N2—C9	−70 (2)	C13—N2—C9—C8	177.8 (2)
O5—Co1—N2—C9	1.2 (2)	Co1—N2—C9—C8	−0.9 (3)
O1—Co1—N2—C9	−92.6 (2)	O6—C8—C9—N2	−178.8 (3)
O3—Co1—N2—C9	95.5 (2)	O5—C8—C9—N2	−0.3 (4)
O7—Co1—N2—C9	178.4 (2)	O6—C8—C9—C10	−0.1 (5)
N1—Co1—N2—C13	111 (2)	O5—C8—C9—C10	178.4 (3)
O5—Co1—N2—C13	−177.5 (2)	N2—C9—C10—C11	1.1 (5)
O1—Co1—N2—C13	88.8 (2)	C8—C9—C10—C11	−177.5 (3)
O3—Co1—N2—C13	−83.1 (2)	C9—C10—C11—C12	−0.3 (5)
O7—Co1—N2—C13	−0.3 (2)	C10—C11—C12—C13	−0.6 (5)
N1—Co1—O1—C1	−7.2 (2)	C9—N2—C13—C12	0.0 (4)
N2—Co1—O1—C1	171.9 (2)	Co1—N2—C13—C12	178.7 (2)
O5—Co1—O1—C1	93.8 (2)	C9—N2—C13—C14	−179.4 (2)
O3—Co1—O1—C1	−24.1 (3)	Co1—N2—C13—C14	−0.7 (3)
O7—Co1—O1—C1	−114.5 (2)	C11—C12—C13—N2	0.8 (5)
N1—Co1—O3—C7	4.2 (3)	C11—C12—C13—C14	−179.9 (3)
N2—Co1—O3—C7	−175.2 (3)	Co1—O7—C14—O8	179.7 (3)
O5—Co1—O3—C7	−96.4 (3)	Co1—O7—C14—C13	−2.4 (4)
O1—Co1—O3—C7	21.3 (4)	N2—C13—C14—O7	2.2 (4)
O7—Co1—O3—C7	112.7 (3)	C12—C13—C14—O7	−177.2 (3)
N1—Co1—O5—C8	176.4 (2)	N2—C13—C14—O8	−179.7 (3)
N2—Co1—O5—C8	−1.4 (2)	C12—C13—C14—O8	0.9 (5)
O1—Co1—O5—C8	99.1 (2)	C20—N3—C16—C17	−0.4 (4)
O3—Co1—O5—C8	−106.7 (2)	C20—N3—C16—C15	−179.5 (3)
O7—Co1—O5—C8	−6.8 (4)	N3—C16—C17—C18	0.8 (5)
N1—Co1—O7—C14	−176.3 (2)	C15—C16—C17—C18	179.9 (3)
N2—Co1—O7—C14	1.4 (2)	C16—C17—C18—C19	−0.7 (5)
O5—Co1—O7—C14	7.0 (4)	C17—C18—C19—C20	0.2 (5)
O1—Co1—O7—C14	−99.9 (2)	C16—N3—C20—C19	−0.1 (5)
O3—Co1—O7—C14	109.3 (2)	C16—N3—C20—C21	178.4 (3)
Co1—O1—C1—O2	−175.8 (2)	C18—C19—C20—N3	0.2 (5)
Co1—O1—C1—C2	6.0 (3)	C18—C19—C20—C21	−178.3 (3)
C6—N1—C2—C3	−1.4 (4)	N3—C16—C15—O10	171.8 (3)
Co1—N1—C2—C3	175.3 (2)	C17—C16—C15—O10	−7.3 (5)
C6—N1—C2—C1	176.7 (3)	N3—C16—C15—O9	−8.7 (4)
Co1—N1—C2—C1	−6.6 (3)	C17—C16—C15—O9	172.1 (3)
O2—C1—C2—N1	−178.4 (3)	N3—C20—C21—O12	−174.5 (3)
O1—C1—C2—N1	0.0 (4)	C19—C20—C21—O12	4.1 (5)
O2—C1—C2—C3	−0.4 (5)	N3—C20—C21—O11	6.2 (4)
O1—C1—C2—C3	178.0 (3)	C19—C20—C21—O11	−175.2 (3)
N1—C2—C3—C4	1.2 (5)	N4—N5—C22—C23	−115.4 (3)
C1—C2—C3—C4	−176.6 (3)	N4—N5—C22—C27	68.4 (4)
C2—C3—C4—C5	−0.2 (5)	C27—C22—C23—C24	−0.1 (5)
C3—C4—C5—C6	−0.6 (5)	N5—C22—C23—C24	−176.3 (3)
C2—N1—C6—C5	0.6 (4)	C22—C23—C24—C25	−0.3 (5)
Co1—N1—C6—C5	−176.1 (2)	C23—C24—C25—C26	0.7 (6)
C2—N1—C6—C7	−179.5 (3)	C24—C25—C26—C27	−0.5 (5)
Co1—N1—C6—C7	3.8 (3)	C25—C26—C27—C22	0.1 (5)
C4—C5—C6—N1	0.4 (5)	C23—C22—C27—C26	0.2 (5)
C4—C5—C6—C7	−179.5 (3)	N5—C22—C27—C26	176.4 (3)

### Hydrogen-bond geometry (Å, °)

**Table:**

*D*—H···*A*	*D*—H	H···*A*	*D*···*A*	*D*—H···*A*
O8—H8···O14	0.82	1.67	2.491 (4)	174
O13—H13A···O6^i^	0.85 (2)	2.29 (2)	2.917 (4)	131 (3)
O13—H13B···O4^ii^	0.85 (3)	2.09 (2)	2.926 (4)	168 (3)
O14—H14A···O4^ii^	0.85 (2)	1.80 (2)	2.645 (4)	174 (3)
O14—H14B···O15	0.85 (3)	1.98 (4)	2.658 (6)	136 (4)
O9—H9···O2^iii^	0.82	1.70	2.520 (3)	173
O11—H11A···O13^iv^	0.82	1.84	2.634 (4)	163
N4—H4A···O6^i^	0.89	2.06	2.935 (3)	167
N4—H4A···O5^i^	0.89	2.53	2.993 (3)	113
N4—H4A···O11^v^	0.89	2.58	3.011 (3)	110
N4—H4B···O10^vi^	0.89	2.05	2.834 (3)	146
N4—H4C···O9^v^	0.89	2.41	2.964 (3)	121
N5—H5A···O1^vii^	0.99 (3)	2.12 (3)	3.060 (3)	158 (3)

Symmetry codes: (i) *x*+1, *y*+1, *z*; (ii) *x*+1, *y*, *z*; (iii) *x*, *y*+1, *z*+1; (iv) *x*, *y*, *z*+1; (v) *x*, *y*, *z*−1; (vi) −*x*+1, −*y*+2, −*z*+1; (vii) *x*, *y*+1, *z*.
